# The lipid–podocyte axis: emerging clues in membranous nephropathy pathogenesis

**DOI:** 10.3389/fmed.2026.1722758

**Published:** 2026-03-04

**Authors:** Sichao Ma, Mingxin Chang, Dongmei Zhang, Yinping Wang, Shoulin Zhang, Hong’an Wang, Yunfan Liu

**Affiliations:** 1 Department of Nephrology, Affiliated Hospital of Changchun University of Chinese Medicine, Changchun, Jilin, China; 2 Office of Research, Affiliated Hospital of Changchun University of Chinese Medicine, Changchun, Jilin, China; 3 The Affiliated Hospital of Changchun University of Chinese Medicine, Changchun, Jilin, China

**Keywords:** cytoskeletal reorganization, lipid–podocyte axis, membranous nephropathy (MN), PLA2R, podocyte

## Abstract

Membranous nephropathy (MN) is an immune-mediated glomerular disease and the most common cause of nephrotic syndrome in adults. Classical paradigms concentrate on the binding of circulating autoantibodies (e.g., anti-PLA2R, anti-THSD7A) to podocytes, resulting in subepithelial immune deposits, complement activation, and podocyte damage. Nonetheless, mounting evidence suggests that lipid metabolism in podocytes is a crucial regulator of MN pathophysiology. Podocyte slit diaphragms are situated within specialized cholesterol-enriched lipid rafts that orchestrate essential structural and signaling complexes. Disturbances in podocyte lipid metabolism (such as excessive uptake or compromised efflux of cholesterol and fatty acids) lead to “lipotoxicity,” marked by mitochondrial oxidative stress, cytoskeletal reorganization, and proinflammatory signaling, ultimately resulting in podocyte hypertrophy, detachment, and apoptosis. This review consolidates recent discoveries regarding the interaction between lipid homeostasis and podocyte biology in minimal change nephropathy (MN). We investigate the interplay between dysregulated lipid profiles, metabolic pathways, and immune injury—specifically, through the promotion of inflammasome activation or the alteration of antigen presentation—and how these interactions may exacerbate glomerular damage. We also talk about translational implications, like how lipid-associated biomarkers (serum lipids, lipidomic signatures, cholesterol-regulatory genes) are related to disease activity and how new therapies (statins, PCSK9 inhibitors, cyclodextrins, nuclear receptor agonists, etc.) might be used to target the metabolic part of MN. The “lipid–podocyte axis” connects podocyte lipid metabolism with immune pathogenesis. This gives us a new way to think about MN and opens up new possibilities for diagnosis and treatment.

## 1 Introduction

Membranous nephropathy (MN) is an antibody-mediated autoimmune glomerulopathy and the primary etiology of nephrotic syndrome in adults. Patients usually show signs of heavy proteinuria, low albumin levels, swelling, and high lipid levels. Kidney biopsies typically exhibit diffuse thickening of the glomerular basement membrane (GBM) accompanied by subepithelial “spike” formation on silver stains. Immunofluorescence microscopy shows small deposits of IgG (mostly IgG4 in primary MN) and complement (C3) along the loops of capillaries. Electron microscopy verifies the presence of numerous subepithelial electron-dense immune complexes and extensive effacement of foot processes in podocytes. The pathogenesis of MN is now understood to center on *in situ* immune complex formation at the podocyte surface. In primary (idiopathic) MN, circulating autoantibodies target intrinsic podocyte antigens, most notably the M-type phospholipase A2 receptor (PLA2R, present in ~60–70% of cases) and, less frequently, thrombospondin type-1 domain-containing 7A (THSD7A) (1). Recently, other antigens, such as NELL-1, exostosins, and semaphorin 3B, have been found in patients who have antibodies but not PLA2R or THSD7A (2). The binding of autoantibodies to podocyte targets activates the complement cascade, especially through the classical and lectin pathways, which leads to the formation of the membrane attack complex (C5b-9) on podocyte membranes. Complement-mediated damage, along with subsequent proinflammatory and pro-fibrotic signaling, compromises the podocyte cytoskeleton and slit diaphragm, resulting in the loss of permselectivity and significant proteinuria. Traditionally, MN has been divided into “primary” (autoimmune) and “secondary” (linked to lupus, cancer, infections, drugs, etc.), but this distinction has become less clear: both primary and secondary forms involve antibodies against podocyte or glomerular antigens and similar injury mechanisms. From an immunological perspective, MN is regarded as a B cell/plasma cell-driven disorder. Anti-PLA2R and anti-THSD7A autoantibodies function not only as biomarkers but also as pathogenic effectors, with their serum titers correlating with disease activity and frequently diminishing during remission (1). These IgG4-dominant antibodies do not fix complement well on their own, but unusual glycosylation and the lectin pathway seem to make the damage worse. The end result is damage to the glomerular filtration barrier caused by complement. Standard treatment focuses on B cells (rituximab, cyclophosphamide) and broadly suppresses immunity to stop the production of autoantibodies. Nonetheless, not all patients exhibit a complete response to immunosuppression, and relapse is prevalent, indicating that supplementary pathogenic factors contribute to podocyte injury in MN. In addition to immunity, lipid homeostasis is increasingly acknowledged as a vital factor influencing podocyte health in minimal change nephropathy (MN). Lipids function not only as metabolic substrates and structural elements but also as dynamic facilitators of cellular signaling. Podocytes have a very complicated structure that depends on lipid-rich microdomains. The slit diaphragm, a specialized intercellular junction, is structured as a cholesterol and sphingolipid-enriched “lipid raft.” Cholesterol is 5–8 times more concentrated in rafts than in the rest of the podocyte membrane. This makes sure that important proteins in the slit diaphragm (nephrin, podocin, NEPH1, CD2AP, TRPC6, etc.) are in the right place and work properly. Fatty acids are also important for the phospholipid bilayers that make up membranes and are the building blocks for bioactive lipid mediators. So, changes in the lipid makeup of podocytes can quickly change the biophysical properties of the filtration barrier and its signaling networks (3). It is now known that too much fat inside cells is bad for podocytes. Dysregulation of fatty acid uptake, triglyceride synthesis, or cholesterol efflux results in ectopic lipid accumulation within podocytes, a condition referred to as lipotoxicity. Experimental and clinical evidence demonstrates that elevated lipid levels (including neutral lipids, cholesterol esters, ceramides, etc.) in podocytes induce mitochondrial oxidative stress, endoplasmic reticulum stress, cytoskeletal disorganization, and proinflammatory signaling (3, 4). These alterations facilitate podocyte hypertrophy, detachment, and apoptotic demise, compromising the glomerular barrier. Podocyte lipid accumulation has been observed in various proteinuric kidney diseases, such as diabetes, FSGS, and MN, and is primarily attributed to intrinsic metabolic defects, including overactive sterol regulatory element–binding protein (SREBP) pathways and downregulated ATP-binding cassette transporters (ABCA1/ABCG1), rather than solely hyperlipidemia in the bloodstream. Lipid-induced inflammasome activation (NLRP3), insulin resistance, and oxidative injury further connect lipid dysfunction with immune signaling in the podocyte. Consequently, a bidirectional crosstalk develops: chronic inflammation (and circulating cytokines) enhance lipid uptake pathways in podocytes, whereas lipid-induced oxidative stress exacerbates local inflammation, establishing a detrimental cycle of injury (3, 4).

Dyslipidemia is a characteristic feature of nephrotic MN; however, recent studies indicate that it reflects and aggravates disease processes rather than serving as a passive bystander. For instance, modified serum lipid profiles and glomerular lipid accumulations are associated with the intensity of proteinuria and renal deterioration. Additionally, particular lipid species and genetic variants in cholesterol pathways are being recognized as prospective biomarkers of MN activity. Podocyte biology is energy-intensive. Podocytes depend heavily on glucose metabolism, but they also have enzymes for breaking down fatty acids and cholesterol, which may be important when the body is under stress. When these metabolic pathways are not working properly (for example, when SREBP activation is higher in people with high levels of lipids), podocytes can be more likely to be hurt when the immune system attacks them. These findings have generated interest in the “lipid–podocyte axis” as a novel aspect of MN pathogenesis, incorporating metabolic and immunological principles.

The goal of this review is to bring together what we know about how lipid metabolism and podocyte biology work together in MN. We summarize the structural and metabolic roles of lipids in podocyte function and emphasize how disruptions in lipid homeostasis may exacerbate podocyte injury in the context of MN. We also look at new evidence that lipid pathways and immune injury can talk to each other. For example, we look at how lipid rafts and palmitoylation affect antibody and complement signaling, and how changing the way immune cells use energy might affect MN. We also talk about the clinical effects: using dyslipidemia and lipidomic markers to keep an eye on the disease and the possibility of lipid-targeted therapies (in addition to regular immunosuppression) to work with current treatments. By combining information from basic, translational, and clinical studies, we hope to show how important metabolic factors are in MN and to help plan future research and treatment for this disease.

## 2 Podocyte biology and function

Podocytes are highly specialized cells critical to the glomerular filtration barrier (GFB) in the kidney, regulating selective filtration through their unique structure and dynamic interactions. Their intricate foot processes, slit diaphragms, and actin cytoskeleton maintain barrier integrity, while their vulnerability to injury underlies proteinuric kidney diseases such as membranous nephropathy (MN). Advances in super-resolution microscopy, multi-omics, and mechanistic studies have elucidated podocyte biology, revealing molecular pathways and therapeutic targets. This section explores podocyte structure, their role in GFB maintenance, and susceptibility to immune-mediated damage in MN, emphasizing their significance in glomerular health and disease.

**Figure 1:**
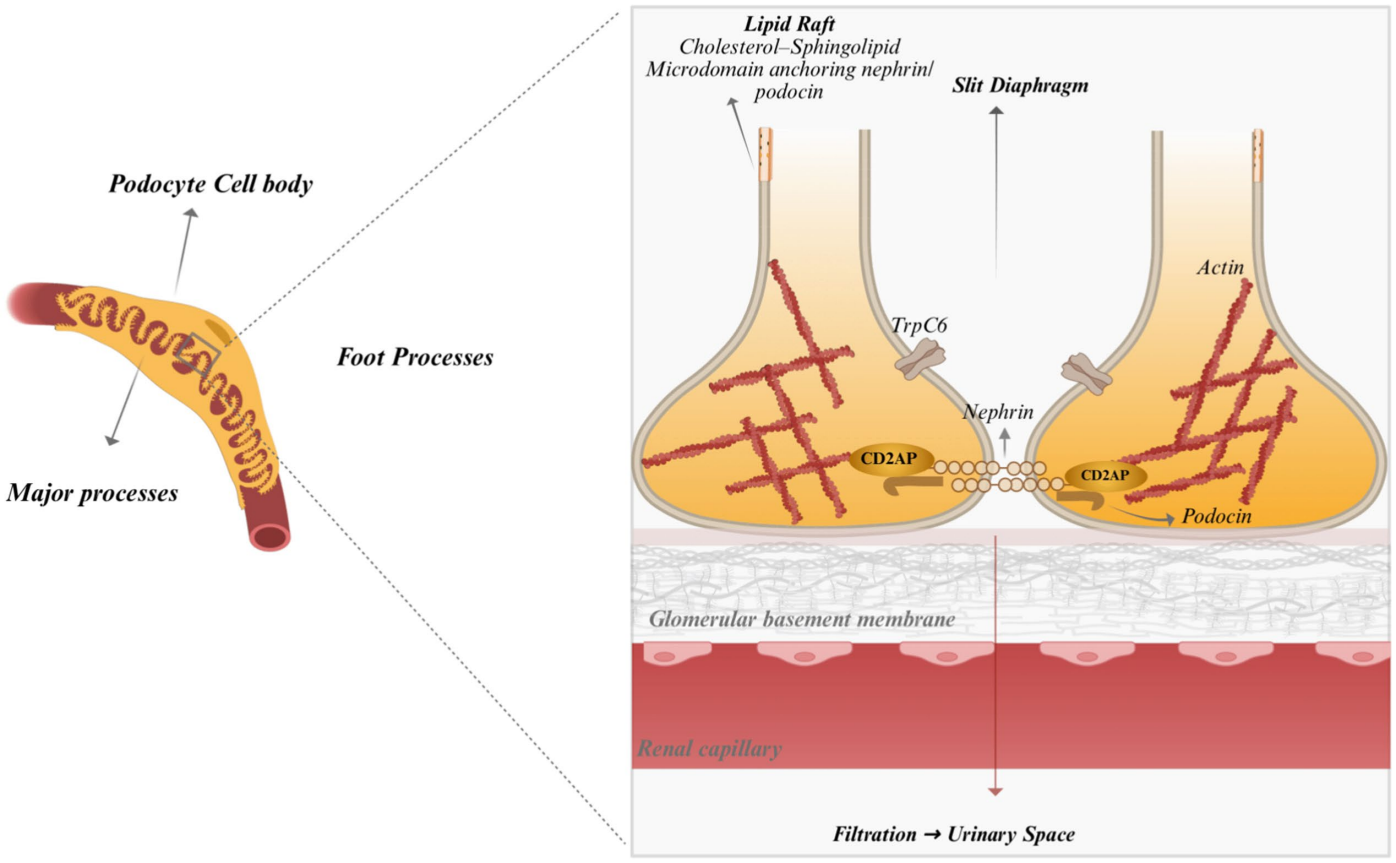
Podocyte structure and lipid organization. The schematic illustrates the architecture of a podocyte resting on the glomerular basement membrane (GBM), including the cell body, major processes, and interdigitating foot processes connected by the slit diaphragm. Lipid rafts within the podocyte membrane serve as signaling microdomains anchoring proteins such as nephrin, podocin, and TRPC6, which link the slit diaphragm to the actin cytoskeleton. These lipid–protein interactions maintain podocyte structure and glomerular filtration barrier integrity. Created with BioRender.com.

The podocyte architecture, including the specialized foot processes and slit diaphragm, is illustrated in Figure 1.

### 2.1 Structure (foot processes, slit diaphragm, cytoskeleton)

Podocytes are specialized epithelial cells in the kidney’s glomerulus, characterized by interdigitating foot processes that form filtration slits essential for the glomerular barrier. Super-resolution microscopy has revolutionized their visualization, achieving resolutions below 100 nm and surpassing traditional electron microscopy (5). Three-dimensional structured illumination microscopy (3D-SIM) enables morphometric quantification of filtration slits in animal models and human biopsies, detecting early changes before proteinuria onset, and integrates with mRNA detection, multiplex staining, and deep learning algorithms (6). Confocal and stimulated emission depletion (STED) microscopy allow imaging in paraformaldehyde-fixed, fresh frozen, and formalin-fixed paraffin-embedded tissues (7). The deep learning-based automatic morphological analysis of podocytes (AMAP) system quantifies foot process morphology, revealing differential effacement patterns across kidney diseases that correlate with proteinuria severity (8).

The slit diaphragm (SD), a junction between foot processes, functions as a molecular sieve comprising proteins such as nephrin, podocin, CD2-associated protein (CD2AP), NEPH1, and transient receptor potential canonical 6 (TRPC6), linked via intrinsically disordered regions (IDRs) (9, 10). Structural modeling shows partner-specific conformational adaptations; mutations such as CD2AP (P532S), podocin (R138Q), and nephrin (G1161V) increase local stability, reduce IDR flexibility, and impair SD assembly (10). Podocin mutations disrupt intraprotein interactions, affecting complex formation with other SD components (11), thereby compromising barrier and signaling functions and leading to proteinuria in nephrotic syndrome (12).

The podocyte cytoskeleton, primarily actin-based, maintains cell morphology via regulators such as synaptopodin, which protects against acute injury by modulating actin reorganization and focal adhesion dynamics. Synaptopodin loss reduces RhoA activity, increases Rac1 activation, and impairs stress fibers and cell migration (13). The cytoskeleton is regulated through SD and focal adhesion hubs, with dysregulation representing a common injury pathway (14). Rho guanine nucleotide exchange factors (e.g., ARHGEF40, ARHGEF2, ARHGEF26) control RhoA and Rac1 to shape podocyte morphology (15). Prototypical Rho GTPases (RhoA, Rac1, Cdc42) regulate actin dynamics in subcellular compartments, which is vital for podocyte health (16).

Podocyte mechanotransduction links the cytoskeleton to barrier integrity, with its unique architecture regulating cell shape, stability, and SD insertion; minor impairments can cause proteinuria (17). TRPC5 and TRPC6 channels mediate calcium influx for cytoskeletal dynamics via nephrin, focal adhesions, and Rho regulation (18). Calcium signaling drives cytoskeletal reorganization in podocytopathies (19). Podocytes adapt to biomechanical stimuli via mechanobiological pathways, with cytoskeletal dynamics and cellular adhesions determining biomechanical resilience (20). Lipid rafts likely support SD organization, with scaffold proteins linking extracellular components to the cytoskeleton, although direct evidence remains limited (12, 21–23).

### 2.2 Role in glomerular barrier

Podocytes are critical for maintaining the glomerular filtration barrier (GFB), which comprises fenestrated endothelial cells, the glomerular basement membrane (GBM), and podocytes to ensure selective filtration (24, 25). Podocytes and endothelial cells engage in bidirectional paracrine and autocrine signaling, with podocytes serving as a central communication hub for GFB development and maintenance (24, 26). In diabetes, hyperglycemia disrupts all GFB layers and this crosstalk, with early endothelial dysfunction including glycocalyx shedding and loss of function driving albuminuria (25, 27). Podocytes secrete key molecules such as vascular endothelial growth factor (VEGF) and angiopoietins, which bind to endothelial receptors to facilitate signal transduction essential for barrier function (28, 29). Additional pathways involve endothelin-1, epidermal growth factor (EGF), semaphorin 3A (SEMA3A), transforming growth factor-β (TGF-β), and C-X-C motif chemokine ligand 12 (CXCL12) (29). These mechanisms preserve glomerular structure under physiological conditions (24, 30).

The glycocalyx forms an additional layer of the GFB; the podocyte glycocalyx, located between foot processes and the GBM, contributes to charge and size selectivity, with its shedding increasing albumin permeability threefold (31). The endothelial glycocalyx, enriched in proteoglycans, glycosaminoglycans, and glycoproteins, restricts albumin passage via a mucinous coat extending into fenestrae (32, 33). Clinical evidence indicates complex relationships between glycocalyx degradation and albuminuria (34). Multi-omics approaches have revealed podocyte-specific molecules essential for barrier function, including pathways related to glycan glycosylphosphatidylinositol (GPI) anchor synthesis, retinol metabolism, and actin regulation; for instance, FARP1 has been validated as crucial (35, 36). These studies also highlight rapid mitochondrial protein synthesis, complex protease networks, and disrupted lipid metabolism, where lipotoxicity induces mitochondrial oxidative stress, cytoskeletal remodeling, and cell death in proteinuric diseases (3, 36). Podocyte metabolism supports barrier integrity through mitochondria, relying on oxidative phosphorylation or glycolysis depending on cellular demands, with shifts occurring during injury (37). Lipid dysregulation establishes a vicious cycle of lipotoxicity, oxidative stress, cytoskeletal remodeling, and inflammation (3, 4), further exacerbating podocyte injury and promoting glomerulosclerosis and fibrosis (38). Under stress, these metabolic disruptions lead to podocyte hypertrophy, detachment, and death, ultimately compromising permselectivity (3).

### 2.3 Podocyte vulnerability in MN

In membranous nephropathy (MN), podocytes are highly vulnerable to immune-mediated injury from autoantibodies such as anti-phospholipase A2 receptor (PLA2R; 50–80% of primary cases) and anti-thrombospondin type-1 domain-containing 7A (THSD7A; 3–5%), which correlate with disease severity and serve as biomarkers (39, 40). Novel antigens include neural epidermal growth factor-like 1 (NELL-1; 5–10% of PLA2R/THSD7A-negative cases), exostosins 1/2, and semaphorin 3B (39, 41). Aberrantly glycosylated anti-PLA2R1 immunoglobulin G4 (IgG4) activates the lectin complement pathway via mannose-binding lectin, leading to C5b-9 formation, proteolysis of synaptopodin and NEPH1, and subsequent cytoskeletal disruption (42). Complement activation, particularly through the membrane attack complex (MAC/C5b-9), drives podocyte damage and proteinuria; the alternative pathway is essential, as demonstrated by the absence of glomerular C5b-9 deposition and albuminuria in factor B-deficient mice (43, 44). Podocytes both produce complement components and are targeted by them, expressing regulators such as CD46, CD55, and CD59 (45). Activation impairs cytoskeletal integrity, resulting in podocyte detachment and proteinuria; moreover, intratubular C5b-9 deposition contributes to progressive kidney injury (46). Injury induces cytoskeletal remodeling and foot process effacement, involving distinct actin networks: contractile myosin IIA-containing cables in cell bodies and major processes, and non-contractile fibers in foot processes. Upon injury, the actomyosin network relocates basolaterally, forming sarcomere-like structures that promote effacement (47). Interactions between the SD and actin maintain barrier integrity; mutations in regulatory proteins disrupt organization, leading to foot process retraction and proteinuria (48). In minimal change disease a related podocytopathy reduced synaptopodin expression correlates with effacement and treatment response (49). Modulation of the cytoskeleton thus presents potential therapeutic targets (50). Mitochondrial dysfunction, oxidative stress, and endoplasmic reticulum (ER) stress are key drivers of podocyte injury, culminating in energy crises, inflammation, and cell death (51, 52). In focal segmental glomerulosclerosis (FSGS), mitochondrial oxidative stress, altered dynamics, and defective biogenesis are associated with genetic mutations (53). ER stress mediates aldosterone-induced podocyte damage through oxidative stress, activating both apoptotic (via CHOP) and protective autophagic responses (54). Biomarkers such as PLA2R and its autoantibodies facilitate diagnosis and guide therapy. Emerging biomarkers encompass proteins, metabolites, noncoding RNAs, and immune cells (55). Podocyturia serves as a hallmark for disease progression (56), while urinary podocyte mRNA enables noninvasive monitoring of activity and therapeutic response (57). Proteomic analyses of biofluids support biomarker discovery, although tissue-based studies remain limited (58). Therapeutic strategies target immune-podocyte interactions, given that podocytes express innate and adaptive immune components (59, 60). Approaches include modulating cytoskeletal dynamics and transcription factors, with sodium-glucose cotransporter 2 (SGLT2) inhibitors and sirtuins promoting repair, alongside paracrine signaling pathways (61). Traditional Chinese medicine offers complement-targeted therapies to mitigate podocyte injury in primary MN (62).

## 3 Lipid homeostasis in podocytes

Podocytes are specialized cells critical to the glomerular filtration barrier (GFB), where they maintain selective filtration through intricate structural and signaling mechanisms. Lipid homeostasis is fundamental to podocyte function, with cholesterol, sphingolipids, phospholipids, and fatty acids playing pivotal roles in membrane integrity, signaling, and energy metabolism. Dysregulation of lipid metabolism, particularly in MN, leads to lipotoxicity, characterized by lipid accumulation, mitochondrial oxidative stress, cytoskeletal remodeling, and inflammation, ultimately causing podocyte injury and proteinuria. Lipid rafts, enriched in cholesterol and sphingolipids, serve as signaling platforms that coordinate slit diaphragm (SD) stability and mechanotransduction, while their disruption exacerbates glomerular diseases such as diabetic kidney disease (DKD) and focal segmental glomerulosclerosis (FSGS). In MN, lipid dysregulation amplifies immune-mediated damage, with autoantibodies targeting podocyte antigens like PLA2R and THSD7A compounding cellular stress. Advances in multi-omics and super-resolution microscopy have elucidated novel lipid regulators and therapeutic targets, highlighting the interplay between lipid metabolism, autophagy, and immune responses. This section reviews the mechanisms of lipid homeostasis in podocytes, the role of lipid rafts in signaling and membrane integrity, and the impact of lipid dysregulation in MN, emphasizing their therapeutic potential.

### 3.1 Cholesterol, sphingolipids, phospholipids, and fatty acids

Podocytes are essential for maintaining the glomerular filtration barrier (GFB) and regulate lipid homeostasis to support their structural and functional roles. Cholesterol homeostasis is tightly controlled through uptake, efflux, and synthesis pathways. The ATP-binding cassette transporter A1 (ABCA1), modulated by the small GTPase Arf6, facilitates cholesterol efflux to maintain cellular lipid balance (63). Pathological conditions disrupt this equilibrium; for instance, angiotensin II reduces ABCA1 expression while enhancing low-density lipoprotein receptor (LDLR)-mediated cholesterol uptake and synthesis via sterol regulatory element-binding proteins (SREBP1, SREBP2) and HMG-CoA reductase (HMGCR), leading to cholesterol accumulation and podocyte injury (64). Tumor necrosis factor (TNF) similarly impairs ABCA1-mediated efflux and sterol-O-acyltransferase 1 (SOAT1)-dependent cholesterol esterification through an NFATc1-dependent mechanism, promoting apoptosis (65). In diabetic conditions, hyperglycemia suppresses Arf6, disrupting ABCA1 recycling and exacerbating cholesterol buildup (63). Sphingolipids, including ceramide and sphingosine-1-phosphate (S1P), contribute to plasma membrane assembly, receptor-effector interactions, and cellular processes such as apoptosis, proliferation, and inflammation (66, 67). Ceramides drive oxidative stress and apoptosis, whereas S1P promotes cell survival and vascular integrity (67). Dysregulated sphingolipid metabolism underlies podocyte injury in nephrotic syndrome, diabetic kidney disease (DKD), and focal segmental glomerulosclerosis (FSGS), with lipid rafts near slit diaphragms (SDs) supporting cytoskeletal dynamics (66, 68). Phospholipids, notably phosphatidylinositol 4,5-bisphosphate (PI(4,5)P2), are critical for SD formation, as demonstrated in *Drosophila* nephrocytes where PI(4,5)P2 depletion abolished SD assembly and reduced endocytosis (69). Phosphatidylserine and phosphatidylinositol enhance protein interactions and membrane fusion, reinforcing SD integrity (70). Nephrin, a central SD component, regulates podocyte adhesion and survival via tyrosine phosphorylation, highlighting phospholipids’ dual structural and signaling roles (71). Fatty acid oxidation (FAO) in podocytes is linked to mitochondrial function, with podocytes relying on mitochondria for energy through oxidative phosphorylation or glycolysis (51). Although tubular epithelial cells depend heavily on FAO, podocytes’ mitochondrial dependence renders them vulnerable to lipotoxicity (72). Dysregulated lipid metabolism induces mitochondrial oxidative stress, cytoskeletal remodeling, and inflammation, leading to podocyte hypertrophy, detachment, and death (3, 38). Multi-omics approaches have identified novel lipid regulators, such as junctional adhesion molecule-like protein (JAML), which modulates SREBP1 signaling via SIRT1, contributing to lipid accumulation in DKD (73). Ceramide-enriched lipoproteins alter sphingolipid metabolism and mTOR signaling, while reduced FAO enzymes (ACOX1, ACOX2, ACOX3) and transporters (ABCD3) exacerbate diabetic nephropathy (74, 75). These findings position lipid metabolism as a key determinant of podocyte function and a promising therapeutic target for proteinuric kidney diseases (3).

### 3.2 Lipid rafts in signaling and membrane integrity

Lipid rafts, cholesterol- and sphingolipid-rich membrane microdomains, are essential for podocyte signaling and structural stability. CD2-associated protein (CD2AP) serves as a critical scaffolding protein, linking nephrin and podocin to the actin cytoskeleton to facilitate bidirectional signaling and maintain slit diaphragm (SD) integrity (76, 77). Tyrosine phosphorylation at CD2AP’s Y10 residue, induced by vascular endothelial growth factor-A (VEGF-A), enhances nephrin binding but may impair GFB function if dysregulated, underscoring the need for precise signaling balance (78). CD2AP also engages PI3K/Akt pathways to promote podocyte survival and prevent apoptosis (77). The SD functions as both a filtration barrier and a signaling hub, with lipid rafts organizing scaffold proteins like CD2AP to coordinate these roles (12). Podocytes face biomechanical challenges from fluid shear stress and pulsatile glomerular forces, necessitating robust mechanotransduction to preserve GFB integrity (79, 80). Pathological conditions, such as hyperfiltration or glomerular hypertension, induce cytoskeletal rearrangement and foot process effacement, leading to podocyte detachment, a primary mechanism of loss in FSGS (80, 81). Lipid rafts facilitate mechanobiological responses by organizing signaling complexes, and their dysregulation exacerbates podocyte dysfunction (20). Therapeutic strategies targeting lipid raft composition, such as cyclodextrins, show promise by mobilizing cholesterol and reducing lipid accumulation in FSGS models, serving as both drug carriers and direct modulators of lipid-related injury (82). Statins and methyl-β-cyclodextrin further disrupt pathological lipid rafts, offering potential to mitigate podocyte damage while preserving normal raft functions (83). These insights highlight lipid rafts’ critical role in podocyte signaling and membrane stability, positioning them as viable therapeutic targets for glomerular diseases.

### 3.3 Lipid dysregulation in membranous nephropathy

In membranous nephropathy (MN), lipid dysregulation drives podocyte injury and proteinuria. Cholesterol accumulation, resulting from impaired efflux mechanisms, is a central pathological feature (Table 1). Downregulation of Arf6 disrupts ABCA1 recycling, leading to cholesterol buildup, particularly in diabetic conditions (63). Sirt6 deficiency exacerbates angiotensin II-induced cholesterol accumulation by impairing ABCG1-mediated efflux, with podocyte-specific Sirt6 deletion worsening kidney injury (84). Altered cholesterol efflux mechanisms are broadly implicated in kidney disease progression, underscoring their role in MN pathogenesis (85). Sphingolipid metabolism is similarly essential, with ceramide accumulation inducing mitochondrial dysfunction and reactive oxygen species production, as evidenced in DKD models where myriocin treatment alleviated ceramide-induced injury and albuminuria (86). Lipoprotein-associated ceramides disrupt mTOR signaling, further compromising podocyte homeostasis (74). Sphingolipids, including ceramide, sphingosine, and S1P, are pivotal in MN, with lipid rafts near SDs supporting filtration function (66, 87). Lipid-mediated oxidative stress amplifies podocyte damage through lipotoxicity, triggering cytoskeletal rearrangement, insulin resistance, and inflammation, which contribute to glomerulosclerosis (4, 38). These interrelated pathways of cholesterol and sphingolipid dysregulation are summarized in Table 1, emphasizing their mechanistic overlap and potential therapeutic targets. In MN, podocytes are targets of autoantibodies (e.g., anti-PLA2R in 70–80% and anti-THSD7A in ~2% of cases) and exhibit immune cell-like properties, participating in innate and adaptive immunity (60, 88). Impaired autophagy exacerbates lipotoxicity by compromising lysosomal clearance, leading to apoptosis and podocyte loss (89). In lupus nephritis, a related condition, autophagy activation protects podocytes, whereas inhibitors aggravate injury (90). Although lipidomics has identified biomarkers in other diseases (e.g., phospholipids in myasthenia gravis and multiple myeloma), specific lipid biomarkers for MN remain underexplored, with current research focusing on proteins and noncoding RNAs (55, 91, 92). Therapeutic approaches, including statins and PCSK9 inhibitors, show variable efficacy in MN; PCSK9 inhibitors reduce LDL cholesterol but may increase nephrotic syndrome risk (93–96). Targeting lipid metabolism and autophagy holds promise for mitigating podocyte injury in MN, but further research is needed to develop specific lipid-based therapies.

**Table 1:** Lipid alterations and pathogenic mechanisms in podocytes in membranous nephropathy (MN).

Lipid class/Pathway	Key molecules/Enzymes	Alterations in MN	Functional consequences in podocytes
Cholesterol metabolism (63, 84, 85)	Arf6, ABCA1, ABCG1, SREBP1/2, Sirt6	↓ Arf6 and Sirt6 → impaired ABCA1/ABCG1 recycling and efflux; ↑ SREBP activation → cholesterol accumulation	Lipid droplet buildup, mitochondrial stress, apoptosis, podocyte detachment
Sphingolipid metabolism (66, 74, 86)	Ceramide, S1P, SPHK1/2	↑ Ceramide, ↓ S1P → oxidative stress, mitochondrial dysfunction	Apoptosis, impaired cytoskeleton, albuminuria
Lipid-oxidative stress axis (4, 38)	ROS, mTOR, Nrf2	Ceramide-driven ROS ↑ → mTOR dysregulation	Cytoskeletal rearrangement, insulin resistance, inflammation
Autophagy–lipid interaction (89, 90)	LC3, p62, lysosomal enzymes	↓ Autophagic clearance → lipid accumulation	Lipotoxicity, apoptosis, podocyte loss
Immune-lipid crosstalk (60, 88)	PLA2R, THSD7A, immune complexes	Autoantibody-triggered signaling → amplified lipid dysregulation	Podocyte injury, proteinuria
Therapeutic modulation (86, 93–95)	Statins, PCSK9 inhibitors, myriocin	Lipid-lowering drugs variably effective; myriocin ↓ ceramide injury	Reduced cholesterol/ceramide burden, partial functional recovery

## 4 Crosstalk between lipids and podocyte injury

**Figure 2:**
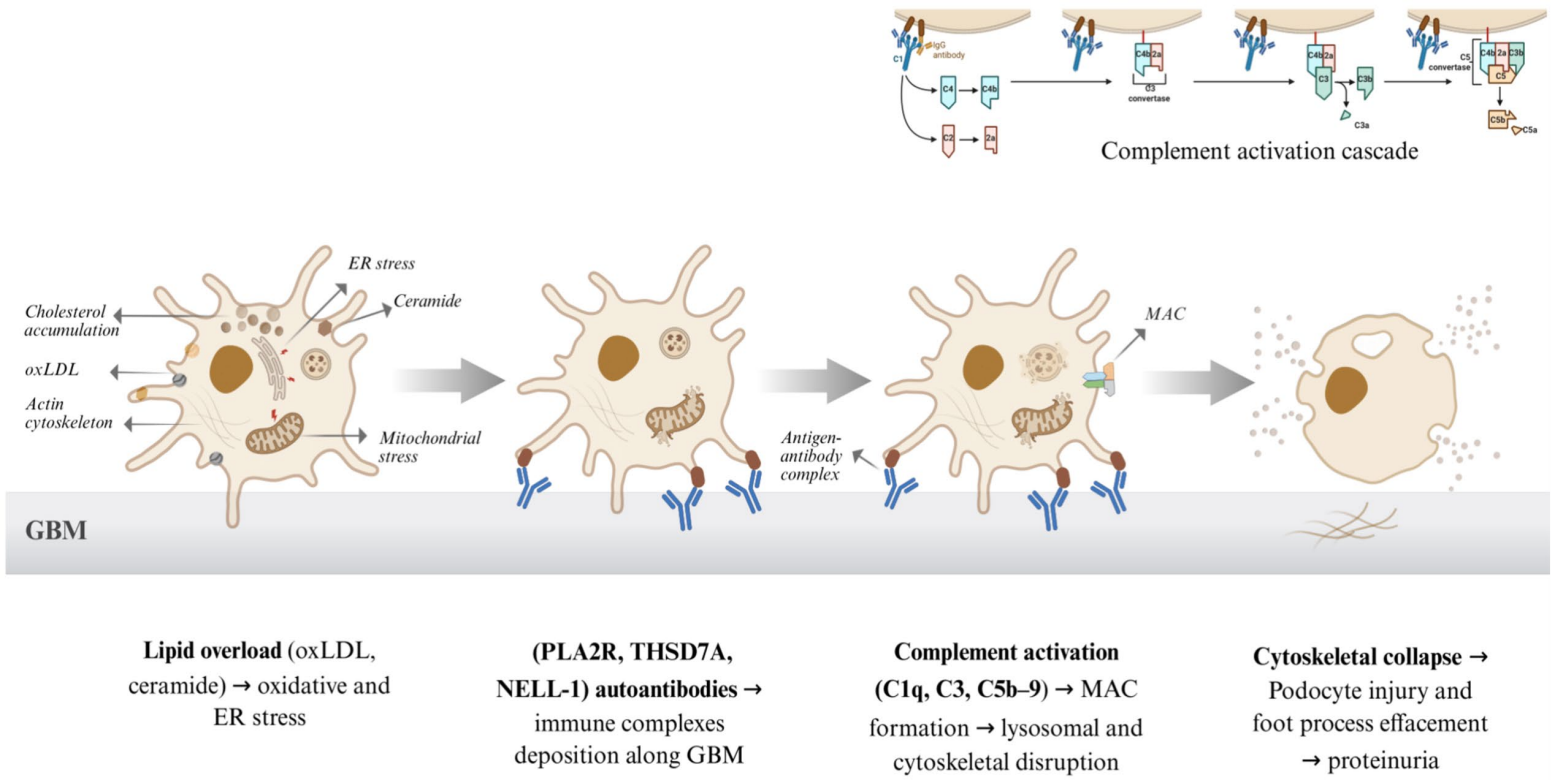
Lipid–podocyte crosstalk in membranous nephropathy (MN). The schematic illustrates the stepwise pathogenic cascade linking lipid dysregulation to immune-mediated podocyte injury. Lipid accumulation and oxidative stress promote immune complex formation on podocyte antigens (PLA2R, THSD7A, NELL-1), triggering complement activation and formation of the membrane attack complex (C5b-9). These events culminate in cytoskeletal disruption, foot process effacement, and proteinuria, defining the core lipid–immune–injury axis in MN pathogenesis. Created with BioRender.com.

Lipid metabolism plays a pivotal role in the pathogenesis of proteinuric kidney diseases, particularly through its impact on podocyte function and glomerular filtration barrier integrity. Dysregulated lipid homeostasis in podocytes contributes to cellular injury, oxidative stress, and inflammation, driving the progression of conditions such as membranous nephropathy (MN) and diabetic kidney disease. This section explores the intricate crosstalk between lipid metabolism and podocyte injury, focusing on lipid rafts as platforms for immune-mediated damage, oxidized low-density lipoprotein (oxLDL) and ceramide-induced apoptosis, dysregulated cholesterol efflux, and lipid-induced oxidative and endoplasmic reticulum (ER) stress leading to cytoskeletal remodeling (Figure 2).

### 4.1 Lipid rafts and immune-mediated podocyte injury

Lipid rafts, specialized membrane microdomains enriched in cholesterol and sphingolipids, serve as critical platforms for autoantibody deposition and complement activation in primary MN, an autoimmune kidney disease characterized by immune complex deposition along the glomerular basement membrane, leading to proteinuria (97). Three major autoantigens phospholipase A2 receptor (PLA2R), thrombospondin type-1 domain-containing 7A (THSD7A), and neural epidermal growth factor-like 1 protein (NELL-1) are large transmembrane glycoproteins expressed by podocytes that elicit IgG4-predominant humoral responses (39, 98). PLA2R-associated MN accounts for 50–80% of primary cases, with autoantibodies targeting a conformational epitope in the N-terminal cysteine-rich ricin domain, while THSD7A and NELL-1 contribute to 1–5% and 5–10% of cases, respectively (39, 99). Confocal microscopy reveals co-localization of IgG and NELL-1 along the glomerular basement membrane, with serum autoantibodies detectable by Western blot (99).

Complement activation is a central pathogenic mechanism in MN, predominantly through the classical pathway, with contributions from the mannose-binding lectin and alternative pathways (100, 101). Mass spectrometry of glomeruli from MN patients shows elevated levels of complement components C1q, C3, C4, C5–C9, and reduced complement regulator CR1, with urinary levels of complement activation products (Ba, C5a, and membrane attack complex) strongly correlating with disease severity (101). Experimental models demonstrate that complement-deficient mice exhibit less severe disease, and C3 silencing attenuates progression, supporting complement-targeted therapies as promising interventions (2, 100). Podocytes express complement components and regulators (CD46, CD55, CD59), yet the C5b-9 complex disrupts autophagy via lysosomal membrane permeabilization, leading to podocyte injury (45, 102). Lipid rafts near slit diaphragms highlight the role of lipid metabolism in podocyte health, and therapeutic strategies targeting raft integrity, such as HDL mimetics, LXR agonists, and cyclodextrins, aim to modulate cholesterol and sphingolipid metabolism to mitigate damage (103). SMPDL3b, a lipid raft enzyme, regulates plasma membrane fluidity, and its dysregulation in diabetic kidney disease impairs insulin receptor signaling, underscoring the therapeutic potential of targeting lipid rafts (104).

### 4.2 Oxidized LDL and ceramide-induced podocyte apoptosis

Podocyte injury in proteinuric kidney diseases is driven by lipid metabolism dysregulation, particularly through the accumulation of oxLDL and ceramides, which promote apoptosis via oxidative stress and inflammatory pathways. Excessive lipid accumulation leads to lipotoxicity, characterized by mitochondrial oxidative stress, cytoskeletal remodeling, and inflammation, culminating in podocyte apoptosis (3, 4). In MN, the long non-coding RNA XIST promotes apoptosis via the miR-217-TLR4 pathway, while aberrant glomerular filtration activates plasminogen to plasmin, upregulating NADPH oxidase and CD36 expression, enhancing cholesterol uptake, and driving apoptosis (105, 106). CD36, a scavenger receptor, facilitates oxLDL and fatty acid uptake, with its expression markedly increased in chronic kidney disease and diabetic nephropathy (64, 107). CD36 inhibition with sulfo-N-succinimidyl oleate reduces lipid accumulation, oxidative stress, and podocyte damage, highlighting its therapeutic potential (108, 109).

Ceramide accumulation is a key mediator of podocyte dysfunction, inducing mitochondrial damage through reactive oxygen species (ROS) production and disrupting mitochondrial integrity (86). Transcriptomic analyses reveal that C16 ceramide-enriched lipoproteins alter mTOR signaling, downregulating phosphorylated proteins in this pathway (74). Sphingolipids, including ceramides, regulate apoptosis, proliferation, and inflammation, with ceramide synthase 6 (CerS6) producing ceramide (d18:1/16:0) that activates the cGAS-STING inflammatory pathway via mitochondrial DNA leakage (67, 110). In diabetic kidney disease, altered ceramide species distribution correlates with disease progression (111). Protective mechanisms, such as autophagy induced by palmitic acid or heme oxygenase-1 via AMPK-dependent pathways, mitigate apoptosis by reducing ROS, while antioxidant therapies like N-acetyl-cysteine suppress harmful autophagy responses, emphasizing the lipid-oxidative stress axis as a therapeutic target (112–114).

### 4.3 Dysregulated cholesterol efflux and podocyte injury

Impaired cholesterol efflux, mediated by ATP-binding cassette subfamily A member 1 (ABCA1), contributes significantly to podocyte injury and kidney disease progression. ABCA1 deficiency leads to mitochondrial dysfunction via altered cardiolipin levels, which can be reversed by reducing cardiolipin peroxidation (115). Tumor necrosis factor (TNF) and angiotensin II reduce ABCA1 expression, increasing cholesterol uptake and synthesis via LDL receptor (LDLr) and SREBP1/2 pathways, resulting in free cholesterol accumulation and apoptosis (64, 65). Inflammatory stress exacerbates lipid droplet accumulation by upregulating LDLr pathway components (SREBP-2, SCAP), promoting epithelial-mesenchymal transition and podocyte injury (116). Therapeutic strategies, such as 5-arylnicotinamide compounds targeting OSBPL7, upregulate ABCA1-dependent cholesterol efflux, normalizing proteinuria and preserving renal function in mouse models (117). Similarly, ApoE mimetic peptides and LXR agonists enhance cholesterol efflux, though clinical challenges like hypertriglyceridemia persist (118, 119). The LXR-ABCA1 pathway is critical for mitigating lipotoxicity, with Dock5 deficiency exacerbating proteinuric kidney disease by modulating LXRα/CD36 signaling (120, 121).

### 4.4 Lipid-induced oxidative and ER stress leading to cytoskeletal remodeling

Lipid dysregulation in podocytes triggers oxidative and ER stress, driving cytoskeletal remodeling and proteinuria. Ectopic lipid accumulation induces lipotoxicity, characterized by mitochondrial oxidative stress, cytoskeletal remodeling, and inflammation, creating a self-perpetuating redox imbalance cycle (4, 38). In MN, complement activation triggers mitochondrial dysfunction and ROS-mediated pyroptosis (122). Lipid bilayer stress activates the unfolded protein response (UPR) via ATF6, IRE1, and PERK, with IRE1 modulating autophagy and lipolysis under lipid perturbation (123). Palmitic acid-induced lipid accumulation promotes ER stress, actin cytoskeleton rearrangements, and slit diaphragm protein alterations, contributing to foot process effacement (124, 125). CD36-mediated fatty acid uptake exacerbates these effects, which can be alleviated by CD36 inhibition (109). The NRF2 pathway, a key regulator of antioxidant responses, mitigates lipid peroxidation and protects podocytes, highlighting its therapeutic potential (126, 127). Emerging strategies target SREBP and HMGCR pathways, mitochondrial dynamics, AMPK activation, and lipid mediators like omega-6 and omega-3 polyunsaturated fatty acids to preserve podocyte structure and function (61, 128).

## 5 Immune–lipid interactions in membranous nephropathy

MN is a leading cause of nephrotic syndrome in adults, characterized by immune-mediated glomerular injury driven by autoantibody deposition and dysregulated lipid metabolism. The interplay between lipid metabolism and immune responses significantly influences disease pathogenesis, involving complex interactions among B cells, T cells, and podocytes. This section explores the intricate relationships between lipid metabolism, inflammatory signaling pathways, and autoantibody-mediated injury via lipid microdomains, highlighting their roles in MN progression and potential therapeutic avenues.

### 5.1 Lipid metabolism and immune responses

MN is an autoimmune glomerular disease defined by immune complex deposition between podocytes and the glomerular basement membrane, primarily driven by autoantibodies targeting podocyte antigens such as phospholipase A2 receptor (PLA2R) in 70–80% of cases and thrombospondin type-1 domain-containing 7A (THSD7A) in approximately 2% of cases (98, 129). These autoantibodies, predominantly IgG4, correlate with disease activity, with circulating plasma cells and anti-PLA2R antibody levels serving as biomarkers of severity (130). Studies reveal specific alterations in B-cell subsets in MN patients, including decreased marginal-zone B cells and non-switched memory B cells, alongside increased plasmablasts, which correlate with proteinuria and anti-PLA2R antibody levels (131). Regulatory B cells are also elevated, suggesting a complex interplay of B-cell responses in disease modulation (130).

While direct links between cholesterol metabolism and B-cell function in MN are limited, broader research underscores cholesterol homeostasis as a critical regulator of immune cell differentiation, activation, and signaling (132). Impaired cholesterol efflux, such as through ABCA1/G1 deficiency in dendritic cells, can induce lupus-like autoimmune phenotypes, including glomerulonephritis, indicating that lipid metabolism dysfunction contributes to autoimmune kidney disease pathogenesis (133). Lipid metabolites further influence T-cell polarization, particularly the Th17/T-regulatory (Treg) cell balance critical to autoimmunity. Cholesterol and long-chain fatty acid biosynthetic pathways regulate RORγ transcriptional activity by generating lipid ligands, directly impacting Th17 differentiation and IL-17 production (134). Sphingolipid pathways, particularly ceramide and glycosphingolipid synthesis, are essential for Th17 differentiation, with serine palmitoyl transferase required for IL-17A and IL-17F expression (135). Plasma membrane lipid composition, including cholesterol and glycosphingolipids forming lipid rafts, modulates T-cell activation and functional phenotypes, offering potential therapeutic targets for controlling aberrant T-cell responses in MN (136).

Immunometabolism governs immune cell function through metabolic reprogramming, with pathways such as glycolysis, fatty acid oxidation, and the Krebs cycle modulating both innate and adaptive immune responses. The mTOR signaling pathway is pivotal, as its inhibition by rapamycin can ameliorate autoimmune inflammation (137). In systemic lupus erythematosus (SLE), abnormal mitochondrial function, lipid metabolism, and mTOR signaling drive disease pathogenesis, with parallels in MN where metabolic reprogramming alters B and T cell survival, differentiation, and function, potentially triggering autoimmune processes (129, 136). Macrophages also exhibit metabolic diversity, with mTOR networks regulating effector functions through cellular metabolism modulation (138).

Primary and secondary MN share common immunological features but also exhibit distinct patterns. Primary MN is characterized by autoantibodies against PLA2R, THSD7A, and NELL-1, with PLA2R positivity in 61.1% of primary cases compared to 22.2% in secondary cases (139). Both primary and malignancy-associated MN show IgG4-predominant responses, with no differences in antigen-specific IgG subclass distribution (140). However, lupus-associated MN lacks glomerular PLA2R deposition, and HBV-associated MN shows PLA2R positivity in only 7.7% of cases, indicating divergent immunological mechanisms (141, 142).

### 5.2 Inflammatory signaling (NF-κB, inflammasome)

Lipid dysmetabolism in podocytes drives proteinuric kidney disease progression through inflammatory mechanisms, establishing a bidirectional relationship where lipid accumulation promotes inflammation, and inflammatory stress exacerbates lipid disorders (3, 38). Inflammatory cytokines, such as IL-1β, upregulate the LDL receptor (LDLr) pathway, including LDLr, SREBP cleavage-activating protein (SCAP), and SREBP-2, leading to lipid accumulation, podocyte epithelial-mesenchymal transition, and injury (116). This lipotoxicity manifests as insulin resistance, mitochondrial dysfunction, and podocyte hypertrophy, detachment, and death, perpetuating a cycle that drives nephropathy progression (3).

The NLRP3 inflammasome is a key mediator of lipid-driven podocyte injury. CD36-mediated lipid accumulation triggers NLRP3 inflammasome activation in podocytes, leading to IL-1β release and podocyte damage in obesity-related glomerulopathy (143). Podocytes express all necessary inflammasome components, contributing to local kidney inflammation (144). The ceramide signaling pathway, via acid sphingomyelinase, regulates NLRP3 inflammasome activation and inflammatory exosome release in podocytes during obesity (145). The adipokine visfatin further mediates NLRP3 inflammasome formation through ASC-dependent mechanisms, increasing caspase-1 activity, IL-1β production, and podocyte dysfunction, characterized by reduced podocin expression and disrupted F-actin fiber arrangement (146).

Lipid rafts serve as critical signaling platforms in podocyte injury. These cholesterol and sphingolipid-enriched membrane domains harbor receptors and regulatory molecules, facilitating signal transduction in immune cells (147). Visfatin stimulation induces NADPH oxidase subunit aggregation in membrane raft clusters, forming redox signaling platforms that enhance reactive oxygen species production and NLRP3 inflammasome activation, leading to podocyte injury (148). The NLRP3 inflammasome, a central pathogenic mechanism in chronic glomerular diseases, produces pro-inflammatory cytokines in both immune and resident cells, including podocytes (149). In lupus nephritis, autoantibody-mediated podocyte damage triggers local immune responses via toll-like receptors and T-cell activation, exacerbating injury (150).

**Figure 3:**
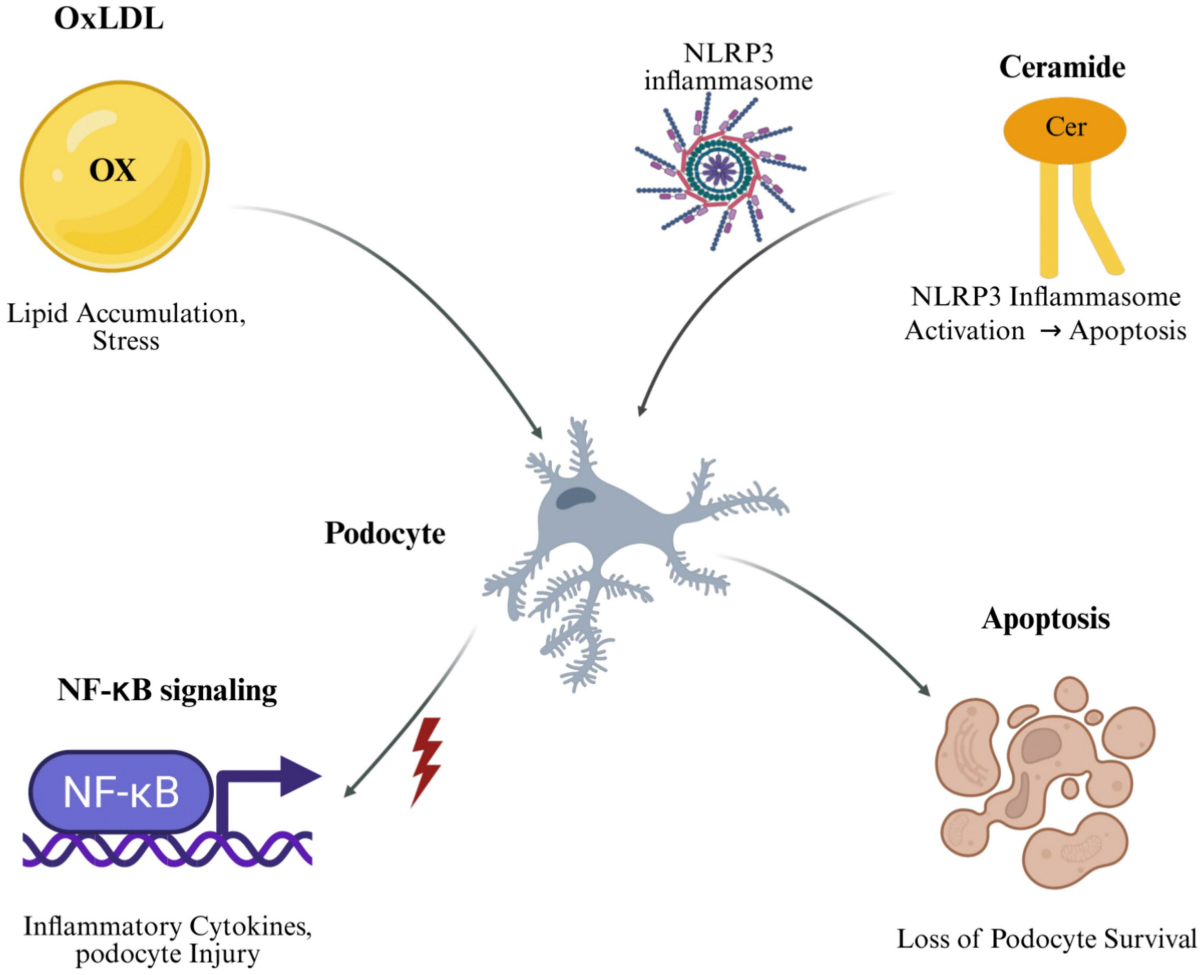
Immune–lipid interactions affecting podocyte survival. OxLDL and ceramide accumulation activate NF-κB signaling and the NLRP3 inflammasome in podocytes, promoting inflammatory cytokine release, oxidative stress, and apoptosis. These immune–lipid pathways form a feedback loop that drives podocyte injury and loss in membranous nephropathy. Created with BioRender.com.

Extracellular vesicles (EVs) facilitate intercellular communication in glomerular pathology. In MN, PM2.5-induced oxidative stress upregulates PLA2R expression in lung tissue, with PLA2R-positive EVs correlating with anti-PLA2R antibody levels (151). Glomerular endothelial cell-derived EVs, enriched with miRNA-200c-3p, induce podocyte dysfunction by decreasing VEGF secretion and increasing mitochondrial stress (152). Podocyte-derived EVs under pathological conditions, such as high glucose, propagate injury to proximal tubular epithelial cells by inducing apoptosis (153). Therapeutic strategies targeting lipid metabolism, NF-κB signaling, and NLRP3 inflammasome activation include statins, PCSK9 inhibitors, cyclodextrins, and FXR/TGR5 dual agonists, which address lipid-mediated kidney injury by reducing inflammation and fibrosis (153–155) (Figure 3).

### 5.3 Autoantibody injury via lipid microdomains

Autoantibody-mediated injury in MN primarily involves circulating autoantibodies against PLA2R and THSD7A, which bind podocyte antigens under nonreducing conditions, inducing IgG4-predominant responses (98, 129, 156). These autoantibodies directly cause podocyte injury and serve as biomarkers for disease monitoring (157). Lipid rafts, enriched with cholesterol and sphingolipids, are critical for Fc receptor signaling. Cholesterol or sphingomyelin depletion impairs IgG-mediated phagocytosis and FcRγ phosphorylation, while lipid rafts recruit signaling proteins to facilitate FcR cross-linking upon immune complex binding (158, 159). In MN, complement activation following antibody deposition triggers C3a/C3aR signaling, contributing to podocyte injury and proteinuria (160). Sphingolipid dysregulation within lipid rafts near podocyte slit diaphragms contributes to cytoskeletal rearrangement, oxidative stress, and inflammation (66, 102). The complement membrane attack complex (C5b-9) exacerbates podocyte injury by impairing lysosome-dependent autophagy, increasing autophagosomes, and promoting lysosomal membrane permeabilization and apoptosis (102). However, C5b-9’s role in tubulointerstitial injury appears limited in selective proteinuria models, suggesting context-dependent effects (161). Protein S-palmitoylation enhances antigen presentation and immune responses. Palmitoylated antigens improve MHC class II-restricted presentation to T cells and MHC class I-restricted CD8^+^ T cell induction, enhancing tumor suppression through efficient lipid bilayer integration (162, 163). S-palmitoylation regulates protein activity, stability, and trafficking in immune cells, with aberrant levels linked to immunologic diseases (164). Lipid raft disruption, using agents like cyclodextrins or statins, reduces autoantibody-mediated cytotoxicity. Cyclodextrins promote cholesterol efflux, ameliorating podocyte injury in preclinical models, while methyl-β-cyclodextrin prevents anti-β2-GPI antibody-induced signaling in endothelial cells (82, 165). Cholesterol depletion with cyclodextrin also mitigates TNF-induced podocyte apoptosis, highlighting cholesterol manipulation as a therapeutic strategy (65).

## 6 Clinical implications

MN, a primary cause of adult nephrotic syndrome, is driven by podocyte injury and lipid metabolism dysregulation, contributing to disease progression and complications like hyperlipidemia. With approximately 30% of MN patients progressing to end-stage renal disease, identifying reliable biomarkers and effective therapies is critical. Dyslipidemia serves as a key biomarker, reflecting disease severity and treatment response, while its correlations with proteinuria and prognosis inform risk stratification. Lipid-lowering therapies, including statins, fibrates, PCSK9 inhibitors, and emerging ANGPTL3 inhibitors, offer potential benefits but require careful consideration due to inconsistent renal outcomes and safety concerns. This section explores dyslipidemia as a biomarker, its correlations with proteinuria and prognosis, and the role of lipid-lowering therapies in MN management.

### 6.1 Dyslipidemia as biomarker

Dyslipidemia is a hallmark of MN, acting as a critical biomarker for diagnosis, monitoring, and risk stratification. Podocyte lipid accumulation induces lipotoxicity, characterized by mitochondrial oxidative stress, cytoskeletal remodeling, insulin resistance, and inflammation, leading to podocyte hypertrophy, detachment, and death (3, 38). This process is exacerbated by a bidirectional interplay between lipid dysmetabolism and inflammation, where inflammatory stress upregulates SREBP-2 and SCAP proteins, enhancing LDL receptor pathway activation and promoting epithelial-mesenchymal transition (116). High-throughput lipidomics studies have identified 105 altered lipids in MN patients, including reduced ceramides, sphingomyelins, diacylglycerols, and phosphatidylcholines following treatment with traditional Chinese medicine. Specific triglycerides, such as TG56:2-FA20:0 and TG56:3-FA20:0, correlate strongly with therapeutic efficacy when combined with clinical parameters, enhancing treatment prediction (4). In broader nephrotic syndrome studies, MN patients exhibit distinct lipidomic profiles, with decreased lysophosphatidylcholine (LPC 16:0;0) and elevated phosphatidylethanolamine and lyso-PE levels compared to controls (166). Genomic analyses further reveal enrichment in cholesterol and arachidonic acid metabolism pathways, with genes like APOA1, APOB, APOC3, CETP, and PLA2G12B associated with circulating lipid concentrations (167). Proteomic studies confirm significant upregulation of lipoprotein-related proteins (APOC1, APOB, APOA1) in idiopathic MN, reinforcing their diagnostic and prognostic utility (168). Anti-PLA2R antibodies, present in 50–80% of primary MN cases, offer high specificity (96.7–100%) but variable sensitivity (25–83%), with tissue-based PLA2R immunohistochemistry (76% sensitivity, 86% specificity) outperforming serum testing (169–171). Emerging biomarkers like NELL-1, exostosin 1, EXT2, and contactin 1 enhance diagnostic precision in PLA2R/THSD7A-negative cases, complementing lipid biomarkers (39). Non-invasive tools, such as gut microbiome analysis (98.36% diagnostic efficiency) and urinary podocalyxin (80.5% sensitivity, 73.5% specificity), further support MN diagnosis (172, 173). These lipid and autoantibody biomarkers enable personalized treatment strategies, improving patient outcomes through targeted interventions.

### 6.2 Correlations with proteinuria/prognosis

Dyslipidemia in MN is closely linked to proteinuria severity and prognosis, serving as a valuable indicator of disease progression and treatment response. Total cholesterol, triglycerides, and LDL levels positively correlate with 24-h urine protein excretion, while total protein and albumin levels show negative correlations, reflecting the impact of nephrotic syndrome on lipid metabolism (174). Hypercholesterolemia independently predicts glomerular PLA2R deposits and serum anti-PLA2R antibody positivity in idiopathic MN, with non-HDL cholesterol exhibiting the strongest correlation with proteinuria levels (175, 176). Patients achieving remission have significantly lower baseline levels of total cholesterol, LDL, and non-HDL cholesterol compared to those with persistent disease, highlighting their prognostic value (176). In nephrotic syndrome, HDL levels are higher in minimal change disease than in MN, while LDL levels are elevated in focal segmental glomerulosclerosis, indicating disease-specific lipid profiles (177). Anti-PLA2R antibody titers are critical prognostic markers, with levels ≤97.6 RU/mL and complete depletion within 6 months predicting spontaneous remission, while epitope spreading at baseline reduces remission likelihood (178–180). In chronic kidney disease (CKD), high total cholesterol and LDL trajectories increase the risk of end-stage renal disease by 21%, and elevated lipoprotein(a) levels (>30 mg/dL) in type 2 diabetes accelerate eGFR decline (2.75 vs. 1.01 mL/min/year) (181, 182). Lipidomic studies in American Indians identified 24 baseline lipids predictive of CKD risk, with longitudinal lipid changes explaining up to 4.8% of eGFR variation, underscoring their prognostic utility (183). In MN, lower HDL levels at diagnosis are associated with progression to end-stage renal disease, and persistent hyperlipidemia in recurrent disease correlates with longer disease duration, particularly in pediatric patients (184, 185). These findings emphasize dyslipidemia’s role as a marker of disease severity and a predictor of renal outcomes, guiding clinical decision-making.

### 6.3 Lipid-lowering therapies

Lipid-lowering therapies, including statins, fibrates, PCSK9 inhibitors, and ANGPTL3 inhibitors, aim to mitigate dyslipidemia in MN, addressing both cardiovascular and renal risks. Statins reduce microalbuminuria and 24-h protein excretion in CKD, with high-intensity statins providing a modest benefit in eGFR decline (0.10 mL/min/1.73 m^2^/year), but they show inconsistent effects on overt proteinuria or CKD progression (186, 187). In diabetic kidney disease, statins decrease albuminuria but do not significantly impact eGFR decline (188). In MN, statins fail to significantly improve lipid profiles at 12-month follow-up, and long-term renal outcomes remain understudied, necessitating further research (177, 189). Fibrates, when combined with statins, reduce acute myocardial infarction risk (HR: 0.77) in moderate CKD but show no significant benefit for major cardiovascular events or mortality, with reversible GFR decreases complicating their use (190, 191). PCSK9 inhibitors effectively lower LDL cholesterol and reduce proteinuria in CKD (57 to 30 mg/g) by preserving megalin function, offering renal benefits in experimental models. However, Mendelian randomization suggests a potential increase in nephrotic syndrome risk, highlighting the need for disease-specific considerations (95, 192, 193). ANGPTL3 inhibitors, such as evinacumab, show promise in nephrotic syndrome models by reducing hypertriglyceridemia, proteinuria, and renal inflammation via inhibition of ROS/GRP78 signaling, though specific MN data are limited (194, 195). Safety concerns in CKD patients include increased risks of statin-induced myalgia (1–10%) and rhabdomyolysis, particularly with worsening kidney function, necessitating careful monitoring (196, 197). Novel therapies targeting the lipid-oxidative stress axis, including SREBP, HMGCR, and AMPK pathways, demonstrate preclinical promise, as do mesenchymal stem cell therapies modulating oxidative stress via GPX1 (4, 198). These approaches require further investigation to optimize efficacy and safety in MN, particularly for personalized treatment strategies tailored to lipid and autoantibody profiles. A comparative summary of current and emerging lipid-targeting and podocyte-protective interventions is provided in Table 2.

**Table 2:** Established and emerging therapeutic interventions targeting the lipid–podocyte axis in membranous nephropathy.

Therapy	Mechanism/Target	Main effects	Renal outcomes/Evidence	Limitations
Statins (177, 186–189)	HMG-CoA reductase inhibition	↓ Cholesterol, ↓ oxidative stress	Mild ↓ microalbuminuria; inconsistent eGFR benefit	Myalgia, rhabdomyolysis
Fibrates (190, 191, 266)	PPAR-α activation → ↑ FA oxidation	↓ TG, ↓ lipid toxicity	↓ AMI risk with statins; reversible ↓ GFR	Caution in CKD
PCSK9 inhibitors (95, 192, 267)	PCSK9 blockade → ↑ LDLR recycling	↓ LDL, ↓ podocyte stress	↓ Proteinuria in CKD; renal protection in models	Possible ↑ nephrotic risk; cost
ANGPTL3 inhibitors (194, 195)	↓ ANGPTL3 → ↑ LPL activity	↓ TG, ↓ inflammation	↓ Proteinuria in nephrotic models	Limited MN data; long-term safety
AMPK/SREBP modulators (4, 198)	Regulate lipid synthesis & stress	↓ Lipotoxicity, ↓ apoptosis	Preclinical benefit	No clinical data
MSC therapy (4, 198)	Antioxidant (GPX1) & immunoregulation	↓ ROS, ↓ podocyte injury	Preclinical promise	Experimental stage

In summary, dyslipidemia in MN is a pivotal biomarker, reflecting disease severity and guiding prognosis through its correlations with proteinuria and renal outcomes. Lipid-lowering therapies offer potential cardiovascular and renal benefits but face challenges due to inconsistent effects and safety concerns in CKD. Integrating lipid biomarkers with autoantibody profiles and emerging therapies targeting lipid metabolism and oxidative stress could enhance personalized MN management, improving outcomes through targeted interventions. Robust clinical validation and careful monitoring are essential to translate these approaches into effective clinical practice.

## 7 Emerging therapeutic perspectives

**Figure 4:**
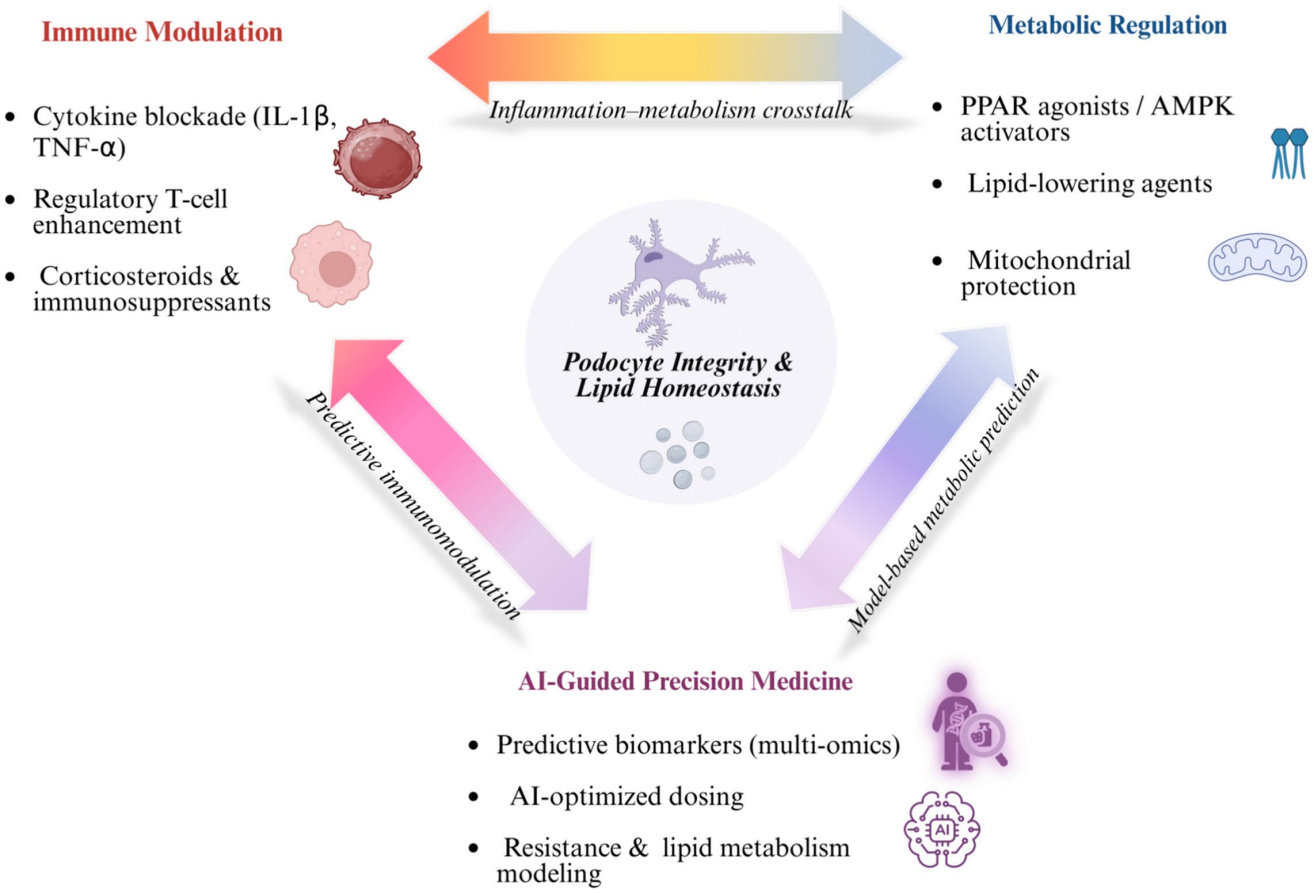
Conceptual schematic illustrating the therapeutic landscape targeting the lipid–podocyte axis. Immune modulation targets cytokine-driven injury via regulatory T-cell enhancement and immunosuppressants; metabolic regulation restores lipid and energy homeostasis through AMPK and PPAR activation; and AI-guided precision medicine integrates multi-omics and predictive modeling to optimize individualized interventions. Inter-domain crosstalk between immune, metabolic, and AI-driven systems converges to preserve podocyte integrity and lipid balance. Created with BioRender.com.

MN, a leading cause of nephrotic syndrome, is driven by autoimmune and metabolic dysregulation, necessitating innovative therapeutic approaches. Emerging strategies include immunosuppressive therapies, metabolic interventions targeting podocyte lipid homeostasis, integrative approaches combining immunomodulation and metabolic support, and artificial intelligence (AI)-driven tools for personalized treatment (Figure 4). This section explores these perspectives, highlighting their potential to transform MN management by addressing autoimmune mechanisms, lipid dysregulation, and the need for precision medicine.

### 7.1 Immunosuppressive therapy

Immunosuppressive therapies, such as rituximab, calcineurin inhibitors (CNIs), and cyclophosphamide, are critical for managing primary MN, particularly in cases linked to anti-PLA2R antibodies. Rituximab, a monoclonal anti-CD20 antibody, demonstrates superior efficacy, with Lu et al. (199) reporting higher total remission rates (68.6% vs. 45.3%, *p* = 0.018) and lower relapse rates (3.4% vs. 38.5%, *p* < 0.001) compared to CNIs in a propensity-matched cohort, alongside reduced eGFR decline (36% in CNI-treated patients had ≥25% decline). Li et al. (200) showed rituximab overcoming CNI dependency, increasing complete remission from 10 to 70% after 12 months in 20 patients. However, a network meta-analysis by Bose et al. (201) found uncertain comparative effects for complete remission between cyclophosphamide, rituximab, and CNIs due to low-certainty evidence. Obinutuzumab, a next-generation anti-CD20 antibody, achieved a 90.0% response rate in rituximab-resistant cases compared to 38.7% for rituximab (*p* < 0.001) (202). PLA2R serostatus influences outcomes, with non-PLA2R-associated MN patients showing higher early remission (76.9% vs. 44.9% at 3 months) and complete remission (30.8% vs. 2.6%) than PLA2R-positive patients, though differences lessen by 9 months (203). Cyclophosphamide and tacrolimus outperform rituximab in PLA2R-negative MN for 12-month complete response rates, but cumulative remission is similar (204). In ANCA-associated vasculitis, rituximab-cyclophosphamide combinations yielded 95% renal survival at 5 years (205), yet the RI-CYCLO trial showed no significant difference in complete remission (16% vs. 32%) or adverse events (19% vs. 14%) between rituximab and cyclophosphamide-corticosteroid regimens in MN (206). Cyclophosphamide may induce faster remission in high PLA2R-Ab cases, though long-term outcomes are comparable (207). Immunosuppressives like cyclosporine increase statin toxicity via cytochrome P450 3A4 interactions, while statins reduce inflammation through the l-mevalonate pathway (96, 208). PLA2R-Ab titers (≤97.6 RU/mL) or depletion within 6 months predict remission, emphasizing the need for tailored immunosuppressive strategies based on biomarkers and resistance profiles (179).

### 7.2 Metabolic interventions targeting podocyte lipid homeostasis

Metabolic interventions targeting podocyte lipid homeostasis address cholesterol and sphingolipid dysregulation in glomerular diseases. Preclinical studies (2019–2024) highlight cholesterol efflux modulation as a protective mechanism. Wright et al. (117) identified 5-arylnicotinamide compounds targeting OSBPL7, upregulating ABCA1-dependent cholesterol efflux, reducing proteinuria, and preserving kidney function in adriamycin-induced nephropathy and Alport syndrome mouse models. Liu et al. (209) showed SOAT1 inhibition reduces cholesterol ester accumulation and lipid droplets in podocytes, enhancing ABCA1 expression and protecting against diabetic kidney disease and Alport syndrome. Sun et al. (210) demonstrated dapagliflozin restores ABCA1 expression via KLF-5, mitigating glucose-induced podocyte apoptosis and cytoskeletal damage. Pressly et al. (211) validated ABCA1 induction through OSBPL7 as a viable therapy for glomerular diseases. PCSK9 inhibitors reduce proteinuria in CKD patients (57 to 30 mg/g and 456 ± 215 to 163 ± 83 mg/g over one year), likely via preserved megalin function, though Mendelian randomization suggests increased nephrotic syndrome risk, necessitating disease-specific considerations (95, 192, 193, 212). Sphingolipid dysregulation, particularly ceramides, drives podocyte apoptosis and CKD progression (67, 68). Ceramide-enriched lipoproteins exacerbate podocyte damage by altering metabolic and signaling pathways (74). Novel interventions, such as nanotechnology-based delivery of dexamethasone/TGFβ1-siRNA or p38α MAPK/p65 siRNA, target lipid-mediated inflammation, offering precise glomerulonephritis treatment (213, 214). These approaches highlight the potential of lipid-targeted therapies, though clinical translation requires addressing disease-specific risks and optimizing delivery systems.

### 7.3 Integrative strategies (immunomodulation + metabolic support)

Integrative strategies combining immunosuppressive and metabolic therapies target both autoimmune and metabolic pathways in MN. Preclinical studies, like Cornaby et al. (215), showed metformin with CTLA4Ig reduced lupus nephritis in mice by suppressing CD4^+^ T cell effector expansion, suggesting potential for MN. Duan et al. (129) highlighted dysregulated B and T cell metabolism in MN, proposing immunometabolic reprogramming as a therapeutic target. Advanced MN modeling, such as GBF-on-chip platforms, facilitates testing these combinations (216). Up to 30% of MN patients fail standard immunosuppression, underscoring the need for novel approaches (217). Lipid metabolism modulation enhances immunotherapy efficacy, as regulatory T cells rely on lipids for immunosuppression (218). In cancer models, targeting the SREBP1-PCSK9 axis with PCSK9 antibodies alongside PD-1 therapy disrupted lipid-mediated immune evasion, offering insights for MN (219). Systems biology approaches, including multi-omics and traditional Chinese medicines like *Tripterygium wilfordii*, reveal metabolic biomarkers and pathways for integrative therapies (220). These strategies could improve outcomes in refractory MN, but clinical translation requires robust validation and standardized protocols (221, 222).

### 7.4 Artificial intelligence in MN

AI and machine learning (ML) are transforming MN management by integrating multi-omics data to predict treatment response and identify therapeutic targets. ML models achieve AUCs of 0.78–0.90 using clinical, imaging, and transcriptomic features to predict outcomes, as shown in related diseases like multiple myeloma (223, 224). In MN, AI-driven multi-omics analysis identifies podocyte-protective targets like EGFR, modulated by phloroglucinol-terpene hybrids via the PI3K/AKT/mTOR pathway in lupus nephritis (225). Liu et al. (4) highlighted the lipid-oxidative stress axis, pinpointing SREBP, HMGCR, and AMPK pathways as key regulators in podocytopathy. Deep learning models using hyperspectral imagery and Raman spectroscopy enhance MN diagnosis with high accuracy (AUC >0.85) by analyzing renal pathology images (226, 227). Multimodal deep learning further improves diagnostic precision (228). Explainable AI (XAI) frameworks like SHAP and LIME increase clinician trust by clarifying predictions, though challenges include data integration complexity and model generalizability (229, 230). Systems biology approaches integrating genomics and metabolomics facilitate biomarker discovery, enabling personalized MN therapies, but robust validation is essential for clinical adoption (231, 232). In summary, emerging therapeutic perspectives for MN, encompassing immunosuppressive therapies, metabolic interventions, integrative strategies, and AI-driven approaches, offer innovative solutions to address autoimmune and metabolic dysregulation. Tailoring treatments based on biomarkers like PLA2R-Ab, optimizing lipid homeostasis, and leveraging AI for precision medicine hold significant promise, though clinical translation requires overcoming challenges in data integration, validation, and equitable implementation.

## 8 Future directions

MN, a leading cause of adult nephrotic syndrome, is driven by autoimmune and metabolic dysregulation, necessitating innovative approaches to improve diagnosis, prognosis, and treatment. Multi-omics integration, longitudinal studies, and novel therapeutics targeting immunometabolic pathways offer promising avenues to advance MN management. These strategies aim to uncover molecular mechanisms, enhance risk stratification, and develop personalized therapies, addressing current limitations in clinical practice.

### 8.1 Multi-omics integration

Multi-omics approaches, combining genomics, transcriptomics, proteomics, lipidomics, and metabolomics, are transforming MN research by elucidating disease mechanisms and identifying biomarkers. The discovery of major antigens like phospholipase A2 receptor (PLA2R) and thrombospondin type-1 domain-containing 7A (THSD7A), alongside minor antigens such as NELL-1, has been facilitated by advanced proteomic techniques, enabling the classification of phenotypically distinct MN subtypes (233). High-throughput technologies, including mass spectrometry and sequencing, have identified diverse biomarkers, including proteins, metabolites, and non-coding RNAs, with diagnostic and therapeutic potential (55). Single-cell multi-omics, particularly lipidomics and transcriptomics, reveal podocyte heterogeneity, identifying metabolically distinct subpopulations vulnerable to lipotoxicity in MN (234, 235). For instance, lipidomics studies highlight disrupted sphingolipid catabolism, ceramide overload, and impaired fatty acid β-oxidation, with biomarkers like urinary hexosylceramide correlating with podocyte structural damage (236). Machine learning enhances multi-omics integration by analyzing high-dimensional datasets, achieving AUCs of 0.78–0.90 for predicting disease progression and reclassifying patients into molecularly defined subgroups (237–239). Systems biology approaches, using mathematical modeling and bioinformatics tools, integrate omics data to uncover novel molecular interactions and therapeutic targets, such as the lipid-oxidative stress axis driving podocyte injury (232, 240). However, challenges include high costs, data integration complexities, cohort heterogeneity, and the need for large-scale validation to translate findings into clinical practice (241, 242). Overcoming these barriers through collaborative research and standardized protocols is essential to leverage multi-omics for precision medicine in MN, improving biomarker discovery and therapeutic development.

### 8.2 Longitudinal studies

Longitudinal studies are critical for understanding dynamic molecular changes and their impact on MN progression and treatment response. Bae et al. (243) demonstrated that lipid ratios (LDL-C/apoB, HDL-C/apoA-1) predict CKD development over 56.5 months, suggesting lipid particle size as a contributor to kidney disease progression. In MN, urinary podocyte-derived microparticles, markers of podocyte injury, decrease post-treatment, linking dynamic changes to disease activity (244). PLA2R autoantibody detection precedes MN diagnosis by months to years, with 66% of seropositive patients showing hypoalbuminemia, highlighting their prognostic value (245). Lipidomic profiling in other diseases, like multiple sclerosis, reveals stage-specific metabolic patterns (e.g., reduced plasmalogens in early stages, increased sphingolipids in progressive stages), suggesting similar approaches could identify MN-specific lipid trajectories (246–248). However, no longitudinal studies directly assess lipid-lowering therapies’ impact on podocyte injury in MN, with existing research focusing on natural disease progression or non-lipid interventions (245, 249–251). Machine learning-based longitudinal modeling, integrating blood biomarkers and electronic health records, outperforms traditional clinical predictors, achieving AUCs of 0.78–0.80 for kidney function decline in high-risk groups (252, 253). Key biomarkers, including tumor necrosis factor receptors and kidney injury molecule-1, reflect inflammation and injury pathways (252). Methodological challenges, such as small sample sizes, high variability, and participant retention (80% at 3 years, 37% at 6 years), limit study reliability (254). Longitudinal lipid variability studies show that intra-individual lipid fluctuations predict clinical outcomes, but measurement uncertainty and biological variability complicate precision (255, 256). Future longitudinal studies in MN should focus on larger cohorts, extended follow-up, and lipid-lowering interventions to establish causal links between dynamic lipid changes and podocyte injury, enhancing prognostic accuracy and therapeutic strategies.

### 8.3 Novel therapeutics

Novel therapeutics targeting immunometabolic pathways offer promising strategies for MN management, addressing podocyte dysfunction and autoimmune mechanisms. The mTOR pathway, a key driver of PLA2R-mediated podocyte apoptosis via PI3K/AKT signaling, is a prime target, with rapamycin showing potential to attenuate podocyte injury (257). Metabolic reprogramming of B and T cells, which drives autoimmune responses in MN, is regulated by mTOR, controlling mitochondrial oxidative phosphorylation, glycolysis, and fatty acid synthesis (129, 258). Lipid-lowering therapies, beyond their cardiovascular benefits, are being explored for podocyte-protective effects. PCSK9 inhibitors reduce proteinuria by preserving megalin function and influence multiple metabolic pathways (LDLR, CD36, VLDL receptors), offering renal protection (259). Drug repurposing, particularly metabolic drugs like metformin, GLP-1 receptor agonists, and SGLT2 inhibitors, shows promise due to their safety profiles and ability to activate AMPK and promote mitophagy, mitigating podocyte lipotoxicity (260, 261). Nanomedicine approaches, such as polymeric nanoparticles delivering dexamethasone or siRNA targeting lipid-mediated inflammation, enable precise podocyte targeting, enhancing therapeutic efficacy (262, 263). Antigen-specific therapies, including pathogenic antibody elimination and chimeric autoantibody receptor T cells, address PLA2R-driven autoimmunity, potentially reducing reliance on nonspecific immunosuppressants like rituximab, which achieves remission in ~66% of patients but fails in up to 30% (129, 217, 264, 265). Targeting the lipid-oxidative stress axis, particularly through AMPK activation, offers a novel approach to disrupt the cycle of lipotoxicity and inflammation (4). Clinical translation requires rigorous trials to validate these therapies’ efficacy and safety in MN, focusing on personalized approaches based on molecular and serologic profiles. In summary, multi-omics integration, longitudinal studies, and novel therapeutics targeting immunometabolic pathways hold significant potential to advance MN management. By elucidating molecular mechanisms, refining prognostic models, and developing targeted therapies, these approaches can enhance precision medicine, improving outcomes for MN patients. However, overcoming challenges like data integration, study design limitations, and therapeutic validation is crucial for clinical implementation.

## 9 Limitations and knowledge gaps

Despite growing interest in the metabolic contribution to membranous nephropathy (MN), several important limitations and knowledge gaps remain that constrain definitive conclusions regarding the role of podocyte lipid dysregulation in MN pathogenesis (268, 269). While this review proposes a unifying “lipid–podocyte axis” framework, it is important to emphasize that the current evidence base is heterogeneous and, in several key areas, remains indirect or extrapolated from related proteinuric kidney diseases. A primary limitation is the relative scarcity of MN-specific mechanistic data directly linking podocyte lipid handling pathways to disease initiation and progression. Classical experimental MN models and human biopsy studies provide convincing evidence for glomerular lipid peroxidation, oxidative stress, and complement-mediated injury, but they offer more limited resolution regarding podocyte-centered lipid pathways, such as sterol regulatory element–binding protein (SREBP) activation, ATP-binding cassette transporter (ABCA1/ABCG1) dysfunction, lipid raft remodeling, or intracellular lipid droplet dynamics. Consequently, several mechanistic nodes discussed in this review—particularly impaired cholesterol efflux, ceramide-driven apoptosis, and defective autophagic lipid clearance—are supported more robustly by data from diabetic kidney disease, focal segmental glomerulosclerosis, and other proteinuric states than from MN-specific systems. While these extrapolations are biologically plausible, they should be interpreted as hypothesis-generating rather than definitive MN mechanisms. A related gap concerns the limited availability of podocyte-resolved lipid data in MN. Most existing studies rely on bulk glomerular analyses, serum lipid profiles, or indirect markers of lipid peroxidation, which do not distinguish lipid alterations occurring specifically within podocytes from those in endothelial, mesangial, or tubular compartments. Given the highly specialized lipid architecture of podocyte slit diaphragms and lipid rafts, this lack of spatial resolution represents a significant obstacle. Advanced approaches such as spatial lipidomics, mass spectrometry imaging, or single-cell multi-omics have not yet been systematically applied to MN biopsies, limiting insight into podocyte-specific lipid remodeling during active disease and remission. Another important limitation relates to experimental model fidelity. Widely used MN models, including passive Heymann nephritis, primarily recapitulate immune complex deposition and complement activation but incompletely reflect the molecular heterogeneity of human MN, particularly PLA2R- and THSD7A-associated disease. As a result, lipid metabolic alterations observed in these models may not fully capture antigen-specific or human-relevant lipid–immune interactions. Moreover, many *in vitro* podocyte studies employ lipid overload or cytokine exposure paradigms that may not accurately reproduce the chronic, immune-mediated metabolic stress characteristic of MN *in vivo*. The lipidomics component in the strict sense remains underdeveloped, representing a major knowledge gap. To date, MN research has largely focused on conventional lipid measurements (total cholesterol, LDL, triglycerides) or selected lipid species, rather than unbiased, high-resolution lipidomic profiling. Comprehensive analyses of cholesterol subclasses, sphingolipid species, phospholipid composition, and fatty acid saturation within glomerular or podocyte compartments are scarce. As a result, it remains unclear which lipid species are primary drivers of podocyte injury in MN, which represent secondary consequences of nephrotic syndrome, and which may serve as reliable disease-specific biomarkers. In the clinical domain, lipidomic biomarkers in MN remain largely exploratory. Although altered lipid signatures have been reported in nephrotic syndrome and in small MN cohorts, these findings are often limited by small sample sizes, cross-sectional design, and lack of external validation. Methodological heterogeneity—including differences in lipid extraction protocols, analytical platforms, normalization strategies, and statistical pipelines—further complicates reproducibility and cross-study comparison. Importantly, many studies do not adequately control for major confounders such as proteinuria severity, renal function decline, statin or immunosuppressive therapy, dietary factors, and systemic inflammation, all of which profoundly influence circulating lipid profiles. Consequently, the specificity of proposed lipidomic biomarkers for MN pathophysiology, as opposed to general nephrotic dyslipidemia, remains uncertain. Another unresolved issue is the temporal relationship between lipid dysregulation and immune-mediated injury (270–272). It is not yet clear whether podocyte lipid abnormalities precede immune complex deposition and sensitize podocytes to antibody- and complement-mediated damage, or whether lipid dysregulation arises predominantly as a downstream consequence of immune injury and proteinuria. Longitudinal studies integrating lipidomics with serologic markers (e.g., anti-PLA2R titers), histologic activity, and clinical outcomes are notably lacking, limiting causal inference. Therapeutically, although lipid-targeted interventions such as statins, PCSK9 inhibitors, cyclodextrins, and sphingolipid modulators show promise, MN-specific efficacy data are limited and sometimes conflicting. Lipid-lowering therapies may improve cardiovascular risk and reduce lipid burden, but their direct impact on podocyte injury, immune activation, and long-term renal outcomes in MN remains incompletely defined. Furthermore, some lipid-modifying agents may exert pleiotropic or context-dependent effects, underscoring the need for disease-specific mechanistic and clinical evaluation. Finally, integration of lipid metabolism with immune signaling in MN—while conceptually compelling—remains insufficiently mapped at the molecular level. How lipid rafts modulate antigen presentation, autoantibody binding, complement activation, and inflammasome signaling in MN podocytes is still poorly understood. Addressing this gap will require coordinated use of advanced imaging, multi-omics, and functional perturbation studies in MN-relevant systems. In summary, while the lipid–podocyte axis provides a useful conceptual framework, substantial gaps remain in MN-specific mechanistic evidence, podocyte-resolved lipidomics, biomarker validation, and causal inference. Addressing these limitations through spatially resolved lipidomics, longitudinal cohort studies, and antigen-specific MN models will be essential to translate metabolic insights into robust biomarkers and targeted therapies for MN (273, 274).

## 10 Conclusion

Recent discoveries have revealed a complicated “lipid–podocyte axis” that interacts with traditional immune systems in MN. Podocyte lipid homeostasis is a vital component in preserving the integrity of the glomerular filtration barrier. Cholesterol-rich lipid rafts support the integrity of the slit diaphragm by organizing nephrin/podocin and other signaling complexes. Podocytes experience lipotoxic stress when lipid handling is disrupted, such as through excessive absorption of free fatty acids or impaired cholesterol efflux. This causes problems with mitochondria, stress in the endoplasmic reticulum, changes to the actin cytoskeleton, and activation of the inflammasome. This kind of metabolic stress makes podocytes more likely to be attacked by the immune system. Complement membrane attack complexes and local cytokines make lipid disturbances worse, which leads to feed-forward injury loops. In clinical practice, dyslipidemia and modified lipid profiles are associated with proteinuria and prognosis in minimal change MN, validating their role as biomarkers of disease activity. Recent research has elucidated that podocyte-specific pathways, such as SREBP-driven lipogenesis, ABCA1/ABCG1 efflux mechanisms, and sphingolipid metabolism, directly affect MN outcomes. Additionally, new “omics” studies are finding lipid types and regulatory genes that could help with diagnosis and figuring out who is at risk. These findings indicate potential therapeutic avenues via metabolic modulation in the future. Instead of just focusing on immunological factors, combination approaches that also bring podocyte lipid metabolism back to normal may work better. Focusing on the lipid-oxidative stress axis is especially promising. For example, blocking SREBP or HMG-CoA reductase to stop too much lipid synthesis, turning on AMPK to boost energy and antioxidant defenses, or boosting ABCA1/ABCG1 to help cholesterol leave the cell. Preclinical investigations have assessed strategies such as statins, cyclodextrins, and FXR/TGR5 agonists, demonstrating protective effects on podocytes. In subsequent MN trials, adjunctive therapies targeting lipoprotein pathways (such as PCSK9 or ANGPTL3 inhibitors) and novel agents that influence cellular lipid flux should be assessed in conjunction with standard immunotherapy. Advanced tools such as lipidomic profiling, single-cell transcriptomics, and machine learning will facilitate more profound phenotyping of metabolic networks and the customization of targeting metabolic nodes. In the end, adding metabolic treatments to MN management could better protect podocyte viability and glomerular function. The lipid–podocyte axis connects immunology and metabolism, giving us a complete picture of how MN develops and how to create new ways to diagnose and treat it (275).
